# Klotho is regulated by transcription factor Sp1 in renal tubular epithelial cells

**DOI:** 10.1186/s12860-020-00292-z

**Published:** 2020-06-22

**Authors:** Yan Li, Yong Liu, Kailong Wang, Yinghui Huang, Wenhao Han, Jiachuan Xiong, Ke Yang, Mingying Liu, Tangli Xiao, Chi Liu, Ting He, Xianjin Bi, Jingbo Zhang, Bo Zhang, Jinghong Zhao

**Affiliations:** Department of Nephrology, the key Laboratory for the Prevention and Treatment of Chronic Kidney Disease of Chongqing, Kidney Center of PLA, Xinqiao Hospital, Army Medical University (Third Military Medical University), Chongqing, 400037 People’s Republic of China

**Keywords:** Sp1, Klotho, Transcriptional regulation, Renal tubular epithelial cells

## Abstract

**Background:**

Klotho is a multifunctional protein, which exists both in a membrane bound and a soluble form. In renal tubules, Klotho is involved in cell senescence, anti-oxidant response, and renal fibrosis, thus regulation of its expression is critical to understand its roles in renal diseases. Indeed, reduced expression was observed in various renal disease. However, the mechanisms underlying transcriptional regulation of the human *klotho* gene (*KL*) largely remain unknown.

**Results:**

Here we demonstrated that the Klotho expression in human renal tubular epithelial cells (RTECs) was enhanced by overexpression of the transcription factor Sp1. On the contrary, Klotho expression was decreased by Sp1 knockdown. Besides, increased expression of Sp1 alleviated TGF-β1-induced fibrosis in HK-2 cells by inducing Klotho expression. Luciferase reporter assays and chromatin immunoprecipitation assays further identified the binding site of Sp1 was located in − 394 to − 289 nt of the *KL* promoter, which was further confirmed by mutation analysis.

**Conclusions:**

These data demonstrate that *KL* is a transcriptional target of Sp1 and TGF-β1-induced fibrosis was alleviated by Sp1 in human RTECs by directly modulating Klotho expression, which help to further understand the transcriptional regulation of Klotho in renal disease models.

## Background

*Klotho* (*KL*) is originally identified by an accidental site defect, which is related to premature multiple organ failure. Owing to alternative splicing, the human *KL* encodes two forms of proteins, which are predominantly expressed in human renal tubular epithelial cells (RTECs). One exists as a full-length membrane-associated form, whereas the other exists as a secreted form lacking the transmembrane segment and the intracellular domain [1]. The membrane Klotho can form a high-affinity co-receptor with fibroblast growth factor (FGF) receptors for FGF23, and thereby contributes to the signal transduction of FGF23 [2, 3]. The secreted Klotho is predominantly detected in cerebrospinal fluid and circulation and is involved in the regulation of anti-oxidative capacity, growth factors pathway and ion transport [4–6].

As known, *KL* is expressed predominantly in kidney, parathyroid gland and choroid plexus [7, 8]. A significantly reduced Klotho was observed in patients with either acute or chronic kidney disease (CKD) [9, 10]. Moreover, *KL*^−/−^ mice experience growth retardation, hypokinesis, osteoporosis, shorten lifespan, while *KL* transgenic mice exhibit increasing resistance to insulin and extending lifespan. Varieties of physiological and pathological factors contribute to the regulation of *KL* expression [4, 9, 10], but the transcriptional regulatory mechanism underlying the *KL* expression is not entirely clear.

Sp1 is a eukaryotic transcription factor highly conserved among mammalian species [11]. It is documented that more than 12,000 Sp1 binding sites have been found in the human genome [12]. Historically, Sp1 has been regarded as a ubiquitous transcription factor responsible for basal expression of housekeeping genes [13]. However, recent studies have revealed that Sp1 is involved in regulating, either inducing or inhibiting transcription of numerous cell type-specific genes [14]. Sp1 can both activate and suppress the expression of genes implicated in senescence, proliferation, differentiation and apoptosis [15], and also involved in inflammation, epigenetic modification and chromatin remodeling [11]. In renal tubular epithelial cells, Sp1 was previously reported to regulate CD2AP promoter activity and expression, suggesting Sp1 is functional in RTECs [16]. Lately, it was reported that both Sp1 and Klotho were significantly decreased in hypoxia/reoxygenation (H/R)-injured RTECs and exogenous Sp1 or Klotho could separately function as the protector during H/R injury [17, 18]. Moreover, in LPS-induced inflammation injury, LPS could down-regulated Sp1-mediated gene transcription, while Klotho was significantly reduced during LPS-induced injury [19, 20]. Further bioinformatics analysis showed no typical TATA or CAAT boxes were found in the human *KL* promoter. Instead, 5 potential Sp1 binding sites were predicted [8]. Thus, we assume that there may be some causative linkers between Klotho and Sp1. However, transcriptional regulation of *KL* by Sp1 has still not been reported.

As is reported, TGF-β1 has been participated in renal fibrosis through inducing epithelial-to-mesenchymal transition (EMT) in RTECs [21]. Evidences also support that EMT has been demonstrated to lead to renal fibrosis [22], the final common pathway to end-stage kidney disease (ESRD). Previous study reported that TGF-β1 could decrease Klotho protein expression, while as a therapeutic target, elevated Klotho expression could inhibit TGF-β1-induced EMT [23].

In the study, we investigated whether Sp1 could affect Klotho expression and TGF-β1-induced fibrosis in human RTECs. Moreover, we further detected the underlying mechanism of regulation of Klotho expression by Sp1.

## Results

### Overexpression of Sp1 dose-dependently induced Klotho expression in human kidney cells

**Fig. 1:**
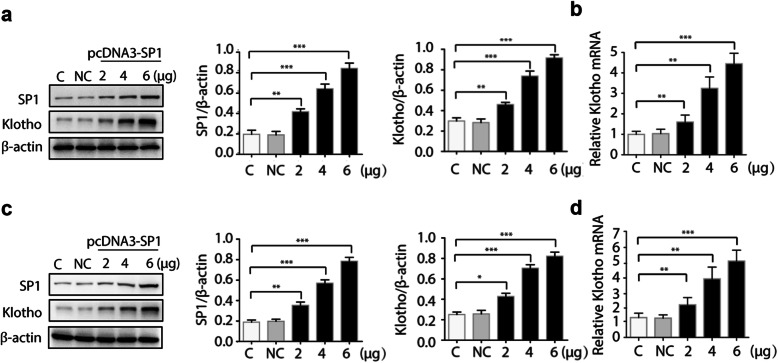
Overexpression of Sp1 upregulates Klotho expression. HK-2 cells (**a**-**b**) or HEK-293 cells (**c**-**d**) were transfected with increasing amount of pcDNA3-Sp1 plasmid or empty control. 48 h after transfection, total proteins were isolated and the protein levels of Sp1 and Klotho were detected by Western blots (**a**/**c**). Total cell RNAs were isolated for quantitative RT-PCR to analyze the mRNA level of Klotho (**b**/**d**). Data were expressed as the mean ± SD in three independent experiments. ***p < 0.001, **p < 0.01, *p < 0.05. NC, empty vector; C: untreated control

To examine the specific involvement of Sp1 in regulating Klotho expression, a Sp1 expression plasmid (pcDNA3-Sp1) was transfected into HK-2 cells to introduce overexpression of Sp1 and Klotho expression was measured both at protein and mRNA levels. As shown in Fig. 1a, Sp1 vector could be efficiently expressed in HK-2 cells. Concomitantly, increased Klotho expression both at protein level and mRNA level was observed in a dose-dependent manner (Fig. 1a-b). To further confirm this finding, another cell line was also transfected with pcDNA3-Sp1. Similarly, dose-dependent induction of Klotho expression by Sp1 was also found in HEK-293 cells (Fig. 1c-d). Thus, Sp1 could positively regulate the Klotho expression in human RTECs.

### Knockdown of Sp1 reduced Klotho expression and fibrosis marker in human kidney cells

**Fig. 2:**
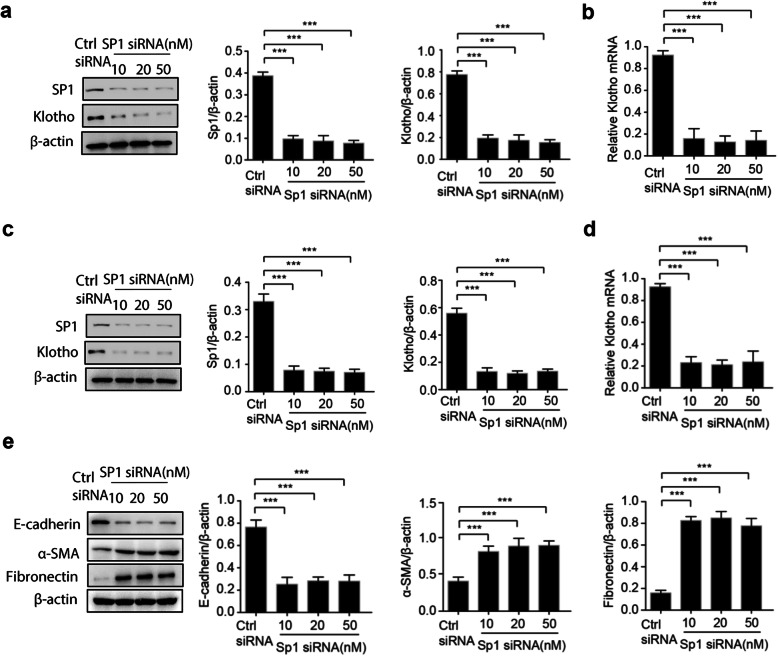
Knockdown of Sp1 reduces klotho expression and induces α-SMA and Fibronectin expression. HK-2 cells (**a**-**b**) or HEK-293 cells (**c**-**d**) were transfected with increasing amount of Sp1-targeted siRNA or control siRNA. 48 h after transfection, total proteins were isolated and the protein levels of Sp1 and Klotho were detected by Western blots (**a**/**c**). Cell total RNAs were isolated for quantitative RT-PCR to analyze the mRNA level of Klotho (**b**/**d**). Total proteins were isolated and the protein levels of E-cadherin, α-SMA, Fibronectin were detected by Western blots (e). Data were expressed as the mean ± SD of three independent experiments. ***p < 0.001. Ctrl siRNA: control siRNA

As overexpression of Sp1 could regulate the Klotho expression, we further wondered the effect of Sp1 knockdown on gene expression of Klotho. Consistently, Sp1 knockdown using a specific siRNA could significantly reduce endogenous Sp1 protein level (Fig. 2a). As expected, suppressed Klotho expression both at protein and mRNA levels was observed in Sp1 knockdown HK-2 cells (Fig. 2a-b). Again, we further repeated the experiment in HEK-293 cells, and the result showed that Klotho expression was noticeably reduced by Sp1 knockdown (Fig. 2c-d). These observations suggested that Klotho expression could be regulated by Sp1 in RTECs.

As reduced Klotho expression was observed in renal fibrosis, we wondered the effects of Sp1 knockdown on renal fibrosis markers. Interestingly, the protein levels of renal fibrosis markers, including α-SMA, fibronectin and E-cadherin, were remarkably altered by Sp1 knockdown in HK-2 cells (Fig. 2e). These demonstrated that Sp1 knockdown could induce renal fibrosis by downregulation of Klotho in RTECs.

### Sp1 correlated with Klotho expression in vivo

**Fig. 3:**
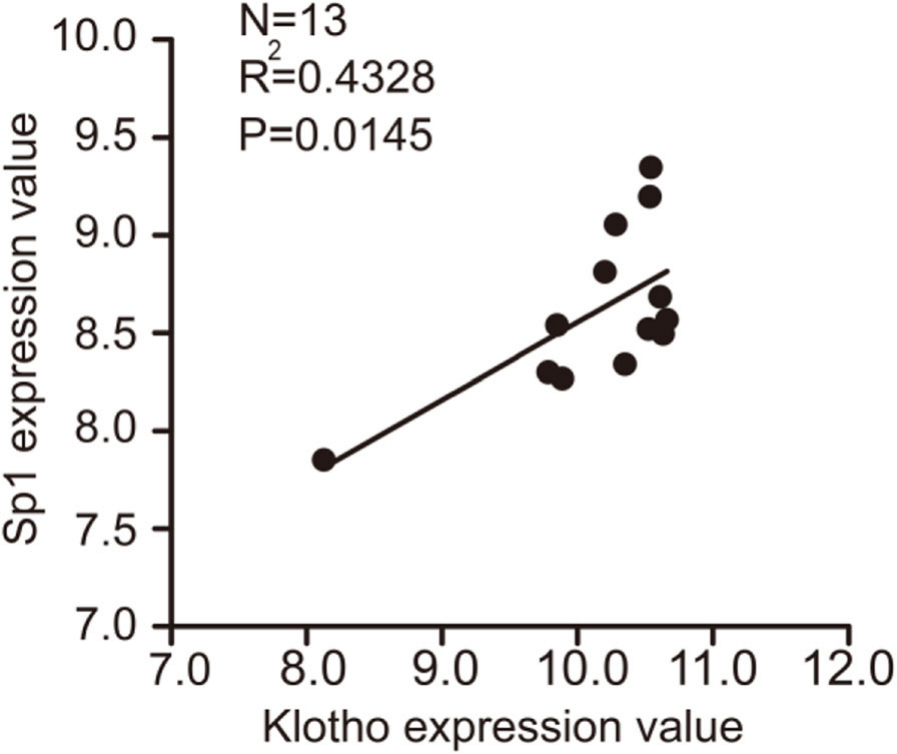
Correlation of Sp1 and klotho mRNA expression level in tubulointerstitial from the healthy human kidney

As known, Klotho is predominantly expressed in human renal tubules. To further investigate the relationship between the Sp1 level and the Klotho expression in vivo, GSE32591 dataset, which contains the gene expression data of micro-dissected human tubulointerstitial compartments from cortical tissue segments of the healthy human kidney, was selected. Here we found that the mRNA expression level of Sp1 in human tubulointerstitial from cortical tissue of health kidney was positively correlated to mRNA expression level of Klotho (Fig. 3). These data suggested that Sp1 might regulate Klotho expression in vivo.

### Overexpression of Sp1 alleviated TGF-β1-induced fibrosis in HK-2 cells

**Fig. 4:**
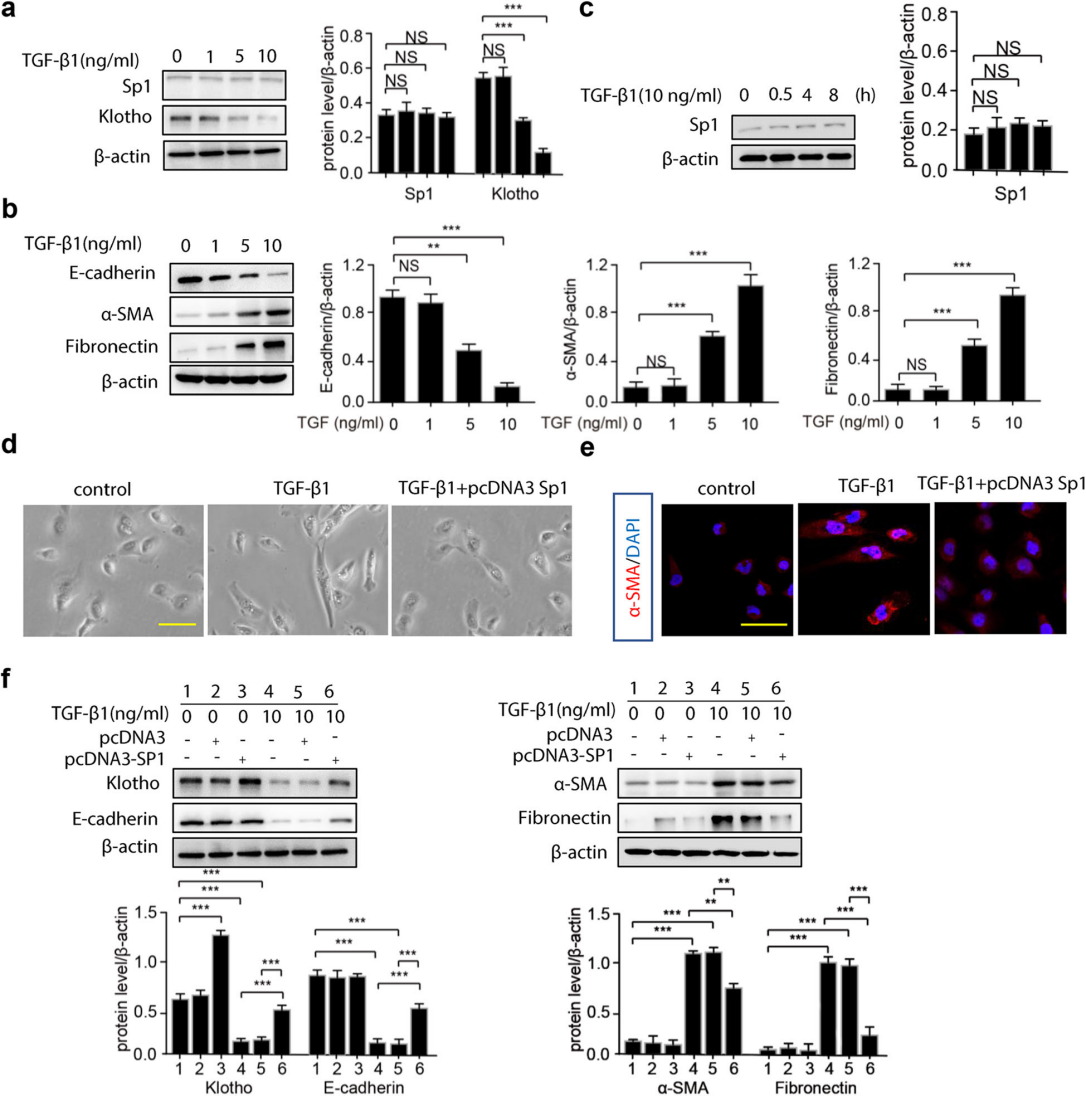
Sp1 recovers morphological and the expression of E-cadherin, α-SMA, Fibronectin of TGF-β1-induced HK-2 cells. HK-2 cells (**a**-**b**) were treated with increasing amount of TGF-β1. (**a**-**b**) 48 h after treatment, total proteins were isolated and the protein of α-SMA, E-cadherin, Fibronectin, Sp1 and Klotho were detected by Western blots. (**c**) HK-2 cells were treated with TGF-β1(10 ng/mL) for 0.5, 4, 8 h, and the protein of Sp1 was detected by Western blots. (**d**-**e**) HK-2 cells were transfected with pcDNA3-Sp1 plasmid followed by treating with TGF-β1 for 48 h, effects of pcDNA3-Sp1 on morphology and α-SMA expression of HK-2 cells. (**f**) pcDNA3-Sp1 plasmid or empty vector were transfected into HK-2 cells followed by treating with TGF-β1. 48 h after treatment, total proteins were isolated and the protein levels of Klotho, E-cadherin, α-SMA, and Fibronectin were detected by Western blots. Data are means ± SD, ***p < 0.001, **p < 0.01. NS, statistically nonsignificant

In HK-2 cells, we found that TGF-β1 treatment had no effect on Sp1 expression (Fig. 4a), whereas it dose-dependently increased the expression of α-SMA and fibronectin, and decreased Klotho and E-cadherin expression (Fig. 4b). Further, we also found that short-term treatment of TGF-β1 had no effect on Sp1 expression in HK-2 cells (Fig. 4c). After treatment with TGF-β1, the morphology of HK-2 cells had changed from normal to shuttle shape. However, the morphological changes were alleviated by pre-transfection with a Sp1 expression plasmid (Fig. 4d). In addition, decreased expression of Klotho and E-cadherin, and increased expression of fibronectin and α-SMA after the TGF-β1 treatment could be partially reversed by Sp1 pre-transfection into HK-2 cells (Fig. 4e & f). The data suggested that Sp1 overexpression could remarkably suppress TGF-β1-induced fibrosis in HK-2 cells.

### Defining the Sp1-responsive region in Klotho promoter

**Fig. 5:**
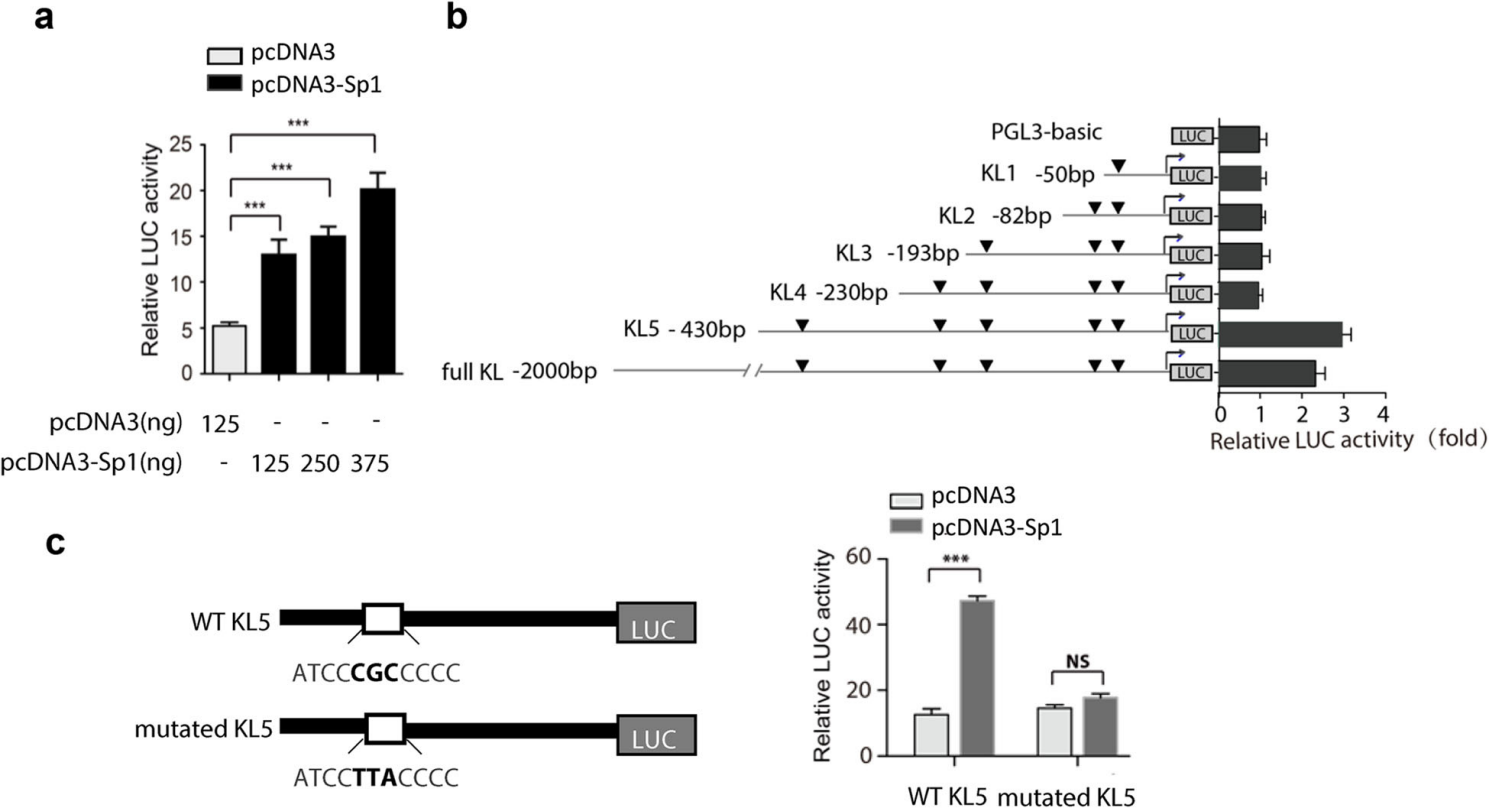
Sp1 binds to the human Klotho gene (*KL*) promoter. (**a**) HEK-293 cells were co-transfected with Full KL/LUC reporter and pcDNA3 or pcDNA3-Sp1 plasmid. 24 h after transfection, cells were lysed and transcriptional activity was detected by dual-luciferase assays. (**b**) Cells were transfected with full length or truncated reporter, combined with pcDNA3 or pcDNA3-Sp1 plasmid. 24 h after transfection, HEK-293 cells were lysed and transcriptional activity was detected. (**c**) HEK-293 cells were transfected with KL5/LUC or mutated KL5/LUC reporter, and transcriptional activity was detected as before. As an internal control, Rinella (pRL-TK) was used to normalize the transfection efficiency. Mean ± SD of triplicate experiments performed independently. ***p < 0.001. NS, statistically nonsignificant

Then we wondered whether Sp1 binding sites existed in Klotho promoter. Bioinformation analysis of human putative Klotho promoter showed that there were 5 potential binding sites (GC boxes) for Sp1 binding in this region. Reporter assay of Full KL/LUC showed that expression vector pcDNA3-Sp1 dose-dependently enhanced the relative luciferase activity (Fig. 5a), suggesting that Sp1 could regulate the transcriptional activity of *KL* promoter in HK-2 cells. Then, a series truncated reporter was constructed to define the Sp1-responsive region (Fig. 5b). To test the inducted activity by Sp1, pcDNA3-Sp1 or control was co-transfected into HK-2 cells with these reporters. Interestingly, induced reporter activity by Sp1 was only detected in *KL5/LUC* construct, which contained − 430 nt to + 8 of Klotho promoter. Its induction by Sp1 was similar to that of full-length Klotho promoter. Compared with non-responsive construct *KL4/LUC, KL5/LUC* construct contained one additional putative binding site of Sp1. To further confirm the involvement of this binding site, the consensus sequence was mutated by site-specific mutation. As expected, no induced reporter activity by Sp1 was observed in the mutated reporter (Fig. 5c).

**Fig. 6:**
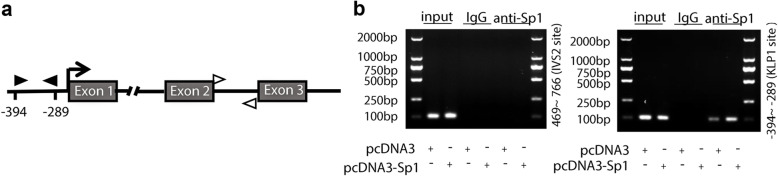
ChIP assay of Sp1 protein binding to the *KL* promoter. (**a**) Schematic diagram of *KL* promoter and control region. Black (target) and white arrow (off target) indicate the position of the ChIP PCR primers. The transcriptional initiation site (arrow) corresponding to the promoter region is considered to be + 1. (**b**) HK-2 cells were transiently transfected with pcDNA3-Sp1 plasmid or empty control (pcDNA3). 24 h after transfection, cells were cross-linked, sonicated and immunoprecipitated with Sp1 antibody or IgG (negative control). The immunoprecipitated DNA and the input DNA were obtained for PCR testing

Finally, ChIP analysis was performed to further confirm the binding of Sp1 in Klotho promoter. After immunoprecipitation with specific antibody against Sp1, the DNA fragments were detected by PCR with primers spanning − 394 and − 289 nt of the *KL* promoter (Fig. 6a). As expected, positive bands were appeared in Sp1 antibody- immunoprecipitated DNA sample, but not in IgG group. In addition, more tensive band was detected in Sp1 transfected HK-2 cells, compared with that in empty vector transfected cells **(**Fig. 6b**)**. Taken together, Sp1 could bind with the Klotho promoter directly.

## Discussion

In this study, we described a transcriptional mechanism underlying the gene regulation of *KL*, a crucial aging-suppressor gene. Klotho expression was enhanced by Sp1 overexpression, while inhibited by Sp1 knockdown. Besides, we found that increased expression of Sp1 in human RTECs attenuated TGF-β1-induced fibrosis by increased expression of Klotho. More importantly, we identified the specific Sp1 binding site within the *KL* promoter region in HK-2 cells.

As a general expressed transcription factor, Sp1 is regarded as a member of the C2H2-type zinc finger family that contains DNA-binding proteins and is highly conserved among mammalian species [24–26]. Sp1 directly bond to GC-rich motifs and is involved in the expression of genes as evidenced by more than 12,000 Sp1 binding sites within the human genome [11, 27]. Sp1 also has been shown to transcriptional regulate a large number of cellular genes critically related to essential cellular functions, including differentiation, senescence, proliferation, and apoptosis [27–29]. In addition, Sp1 could regulate genes with opposing biological functions. For example, Sp1 regulated both oncogenes and tumor suppressor genes, as well as survival-promoting genes and apoptotic-promoting genes [30–32]. However, fully understanding of its roles in various promoters and under various cellular conditions is needed to elucidate contributions to tumorigenesis and other disease processes. The human *KL* promoter, a GC-rich promoter, has 5 potential Sp1 binding sites rather than typical TATA or CAAT boxes [8]. Our luciferase reporter and ChIP assays demonstrated that the specific Sp1-binding site is located between nt − 430 and − 380 in the *KL* promoter region.

It is noteworthy that Klotho is predominantly expressed in RTECs where it regulates senescence, tumor growth, and renal fibrosis [1, 4, 6]. The regulation of Klotho expression is affected by different physiological and pathological conditions [9, 33]. Patients with AKI exhibit dramatically Klotho reduced in kidneys, and scientists identified Klotho acts as an early biomarker in AKI [10]. Furthermore, renal Klotho decreased in mice ischemia reperfusion injury (IRI), while overexpression Klotho played a renoprotective role in mice with ischemia-reperfusion injury (IRI) [10, 18, 34]. Interestingly, scientists reported that the human-induced pluripotent stem cell-derived mesenchymal stromal cells fused with target kidney cells and then delivery Sp1 to protect rat IRI kidney and hypoxia/reoxygenation -injured HK-2 cells, meanwhile Sp1 inhibition totally eliminated the renoprotective role of hiPSC-MSCs-EVs in IRI [17]. To explore the relationship between Sp1 and Klotho, we confirmed that Klotho is positively regulated by the transcription factor Sp1 in cultured human kidney cells.

Regulation of Sp1 expression by TGF-β1 is contradictory in previous research. Some reported that TGF-β1 could upregulate Sp1 expression in rat renal tubular epithelial cells (NRK-52E) [14]. In contrast, several studies observed that after treatment with TGF-β1, only a slight induction or no Sp1 response was observed. Zhou Q et al. found that Fos and Val inhibited TGF-β1 expression, thus upregulated Sp1 and Klotho expression to protect Ang II-induced renal damage in NRK-52E cells [35]. In human dermal fibroblasts, the slight induction of TGF-β1 supplementation on the Sp1 mRNA and protein expression was not statistically significant [36]. Scientists demonstrated that TGF-β1 supplementation had no effect on the expression of Sp1 mRNA or protein in human stellate cells [12]. Therefore, scientists draw the conclusion that TGF-β1-induced Sp1 expression is usually weak and dependent upon cell line. Our results showed that TGF-β1 did not affect Sp1 expression in HK-2 cells.

It has been reported that TGF-β1 promoted renal fibrosis through inducing EMT in RTECs, and this was partially through inhibiting Klotho expression. In our research, when Sp1 was transfected into HK-2 cells, the expression of Klotho was increased, whereas the TGF-β1-induced EMT was alleviated, suggesting that TGF-β1-induced fibrosis could be attenuated by elevated Sp1 expression and Sp1 contributed to the anti-EMT effect through regulating Klotho expression in HK-2 cells.

To explore the mechanisms underlying the regulation of Klotho by Sp1, our luciferase reporter and ChIP assays suggested that Sp1 may differentially modulate klotho expression under specific conditions, although the precise upstream signaling pathways remain to be elucidated. Further, as the Sp1 binding site is relatively far away from the transcription start site, other transcription factors are still needed to be investigated in the regulation of *KL* expression.

## Conclusions

Collectively, our study demonstrated that overexpression of Sp1 alleviated TGF-β1-induced fibrosis in HK-2 cells by inducing Klotho expression and Sp1 directly modulated Klotho expression in kidney cells by binding to a specific CG-rich site between nt − 430 and − 380 in the *KL* promoter region, which help to further understand the transcriptional regulation of Klotho in renal disease models.

## Methods

### Plasmid and reporter gene construct

The coding region of Sp1 was amplified by RT-PCR (primer sequences in Table 1) from HK-2 cells and was inserted into pcDNA3.0 vector to construct recombinant plasmid pcDNA3- Sp1 which were further confirmed by sequencing. The human KL promoter region (− 1900 to + 8) was cloned into the pGL3-basic reporter to yield Full KL/LUC (a gift from Professor Gu Jun, Peking University, China). A series of truncated deletions in the 5′ region of the KL promoter were also constructed by insertion of PCR products into pGL3-basic by homologous recombination, yielding -430KL5/LUC, −230KL4/LUC, −193KL3/LUC, − 82 KL2/LUC, and -50KL1/LUC (the first number of each construct indicates the first nucleotide of the KL promoter, with the transcriptional start site defined as + 1) [17]. To test the binding specificity, the potential Sp1 binding site in KL5/LUC was mutated using a PCR-based site-directed mutagenesis kit (Sangon Biotech, Shanghai, China) (from ATCCCGCCCCC to ATCCTTACCCC). All primers are listed in Table 1.

**Table 1:** Plasmid constructs

Name	Sequence
SP1 forward primer	5′-TCACTATAGGGAGACCCAAGCTTATGGATGAAATGACAGCTGT-3′
SP1 reverse primer	5′-CCGAGCTCGGTACCAAGCTTTCAGAAGCCATTGCCACTGA-3’
KL5 forward primer	5′-TCTGCGATCTAAGTAAGCTTCTCCGAGTGGGAGAAAAGT-3’
KL5 reverse primer	5′-AGTACCGGAATGCCAAGCTTGCTGCGCGGGAGCCAGGCT-3’
KL4 forward primer	5′-TCTGCGATCTAAGTAAGCTTCGGGAGCTGGGAGAAACAG-3’
KL4 reverse primer	5′-AGTACCGGAATGCCAAGCTTGCTGCGCGGGAGCCAGGCT-3’
KL3 forward primer	5′-TCTGCGATCTAAGTAAGCTTTCCCGGGCACCCCTCGCCCT-3’
KL3 reverse primer	5′-AGTACCGGAATGCCAAGCTTGCTGCGCGGGAGCCAGGCT-3’
KL2 forward primer	5′-TCTGCGATCTAAGTAAGCTTTCGCAGGTAATTATTGCCA-3’
KL2 reverse primer	5′-AGTACCGGAATGCCAAGCTTGCTGCGCGGGAGCCAGGCT-3′
KL1 forward primer	5′-TCTGCGATCTAAGTAAGCTTCGGGCATAAAGGGGCGCGG-3’
KL1 reverse primer	5′-AGTACCGGAATGCCAAGCTTGCTGCGCGGGAGCCAGGCT-3’

### Cell culture and treatment

HK-2 (the human renal proximal tubular cell line) and HEK-293 (the human embryonic kidney cell line) obtained from ATCC were separately cultured in DMEM/F12 and DMEM-basic medium with 10% fetal bovine serum (FBS), in a 37 °C incubator containing 5% carbon dioxide. After planked for 24 h, HK-2 cells were treated with TGF-β1 (1 ng, 5 ng, 10 ng/mL) for 48 h.

### Cell transfection

The specific vectors were transiently transfected into the cultured HK-2 or HEK-293 lines using Lipofectamine 2000, a reagent from Invitrogen. SiRNA-based strategies were used to silence endogenous Sp1 expression in HK-2 and HEK-293 cells. Sp1 siRNA sequence was 5′-AUCACUCCAUGGAUGAAAUGATT-3′ [16]. A random scrambled siRNA was used as negative control. Sp1 siRNA was purchased from Gene Pharma. HK-2 and HEK-293 cells were transfected with specific constructs. After transfection, cells were further cultivated until used in subsequent experiments. To further explore the role of Sp1 on HK-2 cells, which were treated with TGF-β1 (10 ng/mL) for 48 h following Sp1 vector were pre-transfected. In each well of 24-well plate, 375 ng for pc-DNA3-Sp1 transfection, 125 ng for full KL, KL5, KL4, KL3, KL2, KL1, PGL-3 basic transfection, and 5 ng for pRL-TK transfection was used for testing relative LUC activity.

### Real-time quantitative PCR

Cell total RNAs from HK-2 and HEK-293 cells was extracted using Trizol (Invitrogen), and cDNA was conducted using a Reverse Transcription Kit (TaKaRa Bio, Japan) following the recommendations of manufacturer. The PCR reaction was then performed in a programmed thermal cycler using a Bio-Rad IQ5 Detection System as previously described. The primer pairs for RT-qPCR are shown in Table 2. The relative expression levels specific genes were based on the 2(−ΔΔCt) approach after normalizing to GAPDH expression.

**Table 2:** Sequence of primers used for real-time quantitative PCR and ChIP-PCR

Name	Forward Primer	Reverse Primer	Size *bp*
Klotho	GCCCACATACTGGATGGTATCAA	ACTGCACTCAGTACACACGGTGA	
β-Actin	ATCTGGCACCACACCTTC	AGCCAGGTCCAGACGCA	
IVS	TTACCAAACGAGAAGCATTACA	CAAAGGGAAGAGGGGACAAG	118
KLP	CTTCTTTGGGCCTCCGAGTG	CGACCAACTTTCCCCGACTT	108

### Western blot analysis

Human HK-2 cells and human HEK-293 cells were lysed and total protein separated by western blot as described previously. The loading protein of HK-2 cells is 20 μg, while 30 μg is for HEK-293 cells. Separated proteins were then transfer to membranes for immunoblotting. Briefly, after blocking, the membranes were incubated using primary antibodies (listed below) at 4 °C overnight. The membranes were washed with TBST buffer for three times, then incubated using secondary antibodies for 1 h at 37 °C. The use of the primary antibodies was as follows: mouse monoclonal anti-β-actin (1:1500, Santa Cruz Biotechnology, USA), rabbit polyclonal anti-Klotho (1:1000, AB181373, Abcam, Cambridge, UK), rabbit monoclonal anti-Sp1 (1:1000, Abcam), rabbit monoclonal anti-E-cadherin antibody (20874–1-AP, Proteintech), rabbit monoclonal anti-α-SMA (ab5694, Abcam, Cambridge, MA), rabbit polyclonal anti-Fibronectin (15613–1-AP, Proteintech) and mouse monoclonal anti-β-actin (1:1000, Santa Cruz). Finally, the signal was detected using ECL plus Reagent as previously described.

### Immunofluorescence assay

To visualize fibrosis marker, after staining with an anti-α-SMA antibody (1:1000), the slides were incubated with fluorescent secondary antibody for 50 min. Then, DAPI was used to stain the nuclei and visualized on a ZEISS confocal microscope.

### Luciferase reporter assay

HEK-293 cells were seeded and co-transfected with Klotho reporters plus the Sp1 expression plasmid using Lipofectamine 2000 reagent. An internal control Rinella luciferase plasmid driven by the TK promoter (pRL-TK) was co-transfected as an internal control. 24 h after transfection, cells were washed in precooled PBS, and samples were prepared in 1 × passive lysis buffer (PLB). The Dual Luciferase Reporter Assay Kit (Promega) was used to detect the relative luciferase activity following the manufacturer’s protocol [18]. Expression was assessed as fold change over control after normalizing for transfection efficiency.

### Chromatin immunoprecipitation (ChIP) assays

ChIP was performed by using a commercially available EZ-ChIPTM kit (Merck Millipore), accoding to the manufacturer’s instruction. HK-2 cells were transfected with pcDNA3-Sp1 for 24 h, harvested, washed with precooled PBS, and then cross-linked by 1% formaldehyde. Cross-linking reactions were halted by addition of glycine. Diluted lysates were prepared and sonicated to yield fragments of 200–500 bp. The sheared cross-linked chromatin fragments were immunoprecipitated with Sp1 antibody or IgG (control) overnight, collected, washed, and reversed cross-fixed at 65 °C. Purified DNA was collected from Spin Columns and used as the template in PCR [18]. Primer sequences designed for two different KL regions are listed in Table 2.

### Availability of supporting data

GSE32591 dataset was selected to further analyze the correlation of klotho and Sp1 in vivo. The dataset we created was rooted in the platform GPL14663 (Afymetrix Multispecies miRNA-a Array). In this dataset, the gene expression data of micro-dissected tubulointerstitial compartments from Cortical tissue segments of healthy human kidney come from the National Center for Biotechnology Information GEO database (https://www.ncbi.nlm.nih.gov/geo/query/acc.cgi?acc=GSE32591).

### Statistical analyses

Data are expressed as mean ± SD. Statistical analysis was analyzed using GraphPad Prism 6.0. Comparison among Groups using t-test or one-way analysis of variance (ANOVA) followed by Tukey’s post hoc tests. Correlation strengths were evaluated by Spearman’s rank correlation coefficient. A *P* < 0.05 (two tailed) was considered statistically significant for all tests. Each experiment was performed at least three replications on independently treated cultures.

## Supplementary information

**Additional file 1.** Western blots in Fig. 1a. The original Western blot images of Sp1、Klotho and β-actin in HK-2 cells transfected with increasing amount of pcDNA3-Sp1 plasmid or empty control.

**Additional file 2.** Western blots in Fig. 1c. The original Western blot images of Sp1、Klotho and β-actin in HEK-293 cells transfected with increasing amount of pcDNA3-Sp1 plasmid or empty control.

**Additional file 3.** Western blots in Fig. 2a. The original Western blot images of Sp1、Klotho and β-actin in HK-2 cells transfected with increasing amount of Sp1-targeted siRNA or control siRNA.

**Additional file 4.** Western blots in Fig. 2c. The original Western blot images of Sp1、Klotho and β-actin in HEK-293 cells transfected with increasing amount of Sp1-targeted siRNA or control siRNA.

**Additional file 5.** Western blots in Fig. 2e. The original Western blot images of E-cadherin, α-SMA, Fibronectin and β-actin in HK-2 cells transfected with increasing amount of Sp1-targeted siRNA or control siRNA.

**Additional file 6.** Western blots in Fig. 4a, b. The original Western blot images of Sp1、Klotho, β-actin, E-cadherin, α-SMA, Fibronectin and β-actin in HK-2 cells treated with increasing amount of TGF-β1.

**Additional file 7.** Western blots in Fig. 4c. The original Western blot images of Sp1 and β-actin in HK-2 cells treated with TGF-β1(10 ng/mL) for 0.5, 4, 8 h.

**Additional file 8.** Western blots in Fig.4f. The original Western blot images of Klotho, E-cadherin, β-actin, α-SMA, Fibronectin and β-actin in HK-2 cells transfected with pcDNA3-Sp1 plasmid or empty vector followed by treating with TGF-β1.

**Additional file 9.** ChIP assay in Fig. 6b. The original images of ChIP assay in HK-2 cells transfected with pcDNA3-Sp1 plasmid or empty vector.

## Data Availability

The datasets used and/or analysed during the current study are available from the corresponding author on reasonable request.

## Abbreviations

RTECs: Human renal tubular epithelial cells
CKD: Chronic kidney disease
IRI: Ischemia reperfusion injury
NRK-52E: Rat renal tubular epithelial cells
EMT: Epithelial-to-mesenchymal transition
ESRD: Final common pathway to end-stage kidney disease
H/R: Hypoxia/reoxygenation
*KL*: The human *klotho* gene
HEK293: Human fetal kidney cells
HK-2: Human proximal renal tubular epithelial cells
