# Supplementary figures and images for: Klotho is regulated by transcription factor Sp1 in renal tubular epithelial cells
Source: BMC Mol Cell Biol. 2020 Jun 22;21:45. doi: 10.1186/s12860-020-00292-z (PMC7309980; doi:10.1186/s12860-020-00292-z)

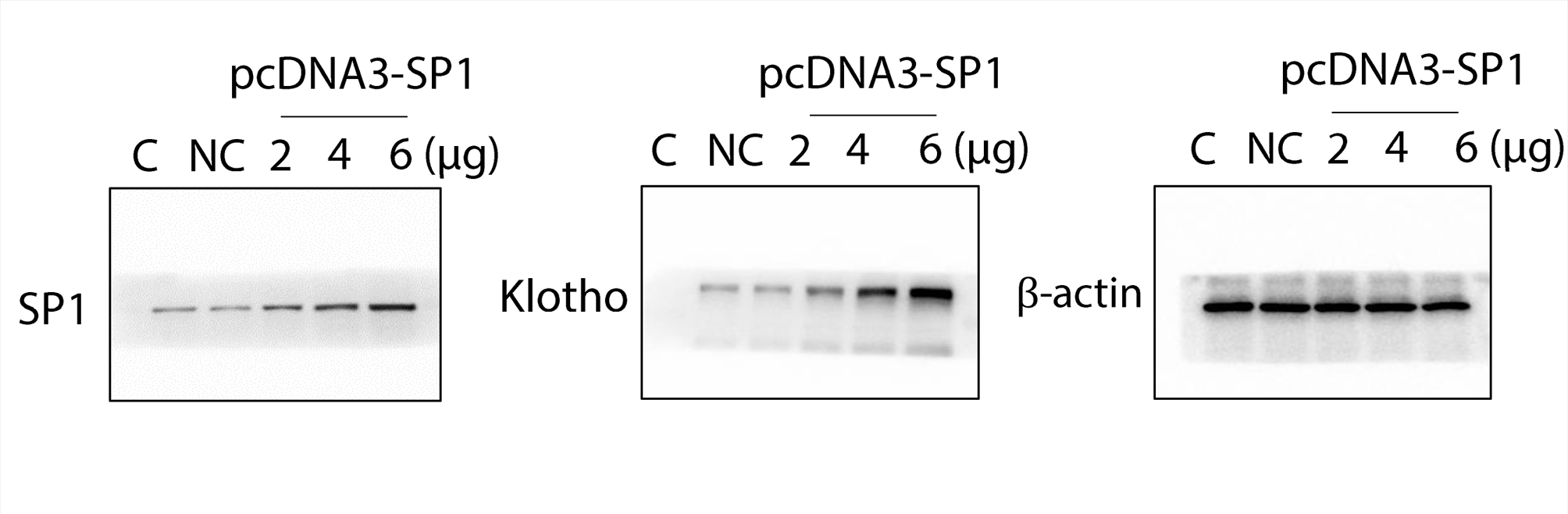

Supplement: Supplementary file 1 — Additional file 1. Western blots in Fig. 1a. The original Western blot images of Sp1、Klotho and β-actin in HK-2 cells transfected with increasing amount of pcDNA3-Sp1 plasmid or empty control. [file 12860_2020_292_MOESM1_ESM.tif]

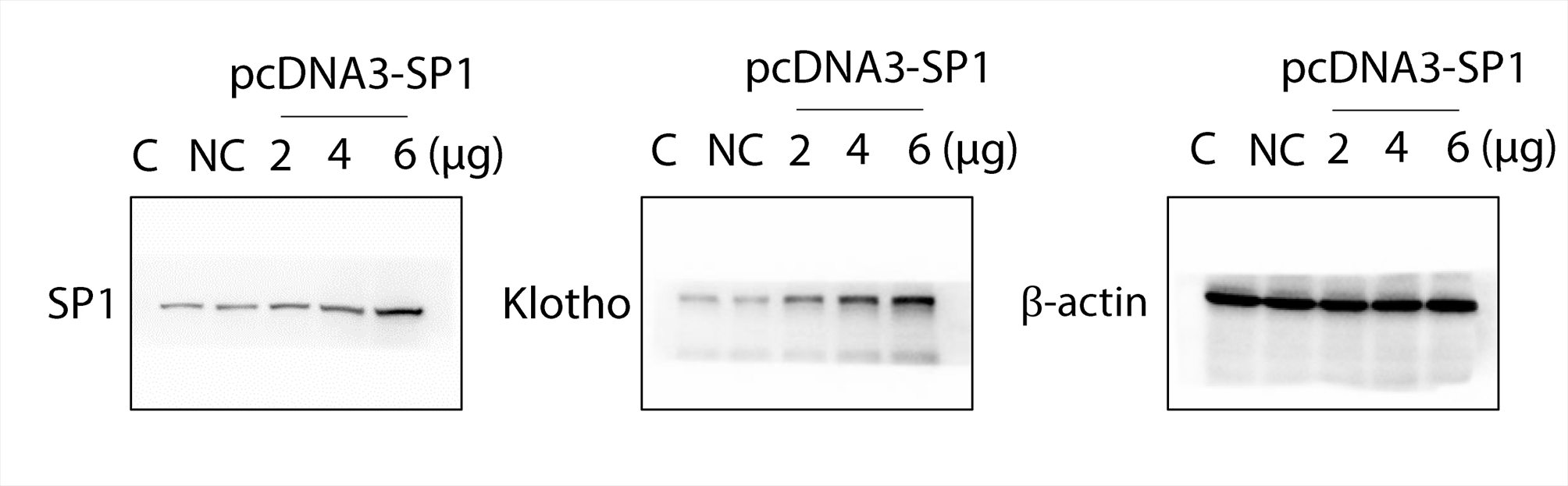

Supplement: Supplementary file 2 — Additional file 2. Western blots in Fig. 1c. The original Western blot images of Sp1、Klotho and β-actin in HEK-293 cells transfected with increasing amount of pcDNA3-Sp1 plasmid or empty control. [file 12860_2020_292_MOESM2_ESM.tif]

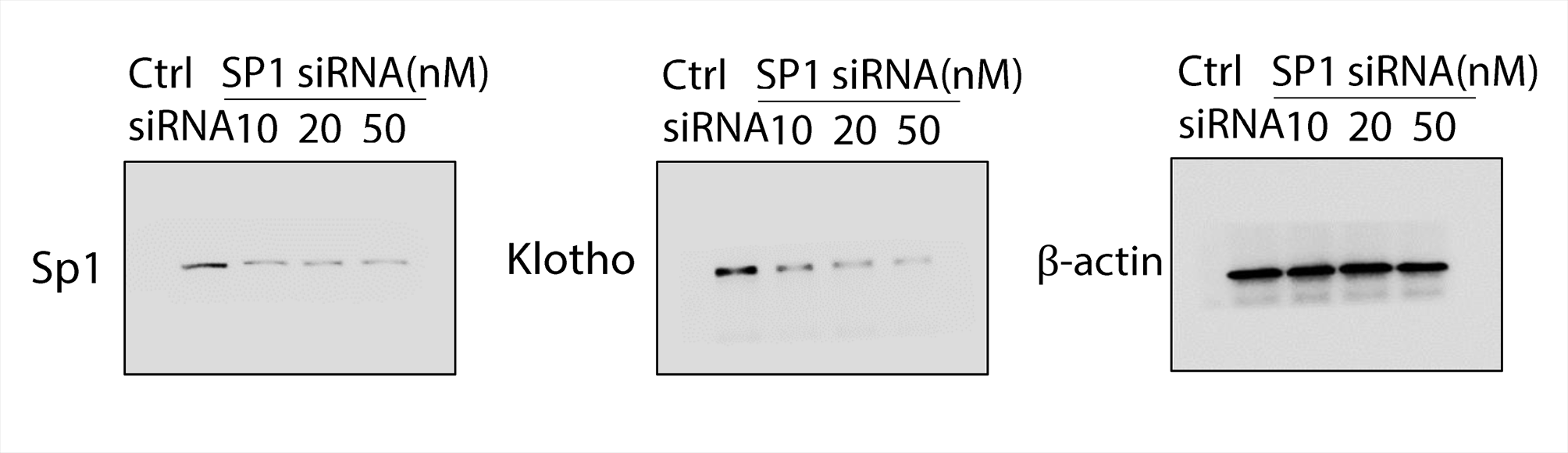

Supplement: Supplementary file 3 — Additional file 3. Western blots in Fig. 2a. The original Western blot images of Sp1、Klotho and β-actin in HK-2 cells transfected with increasing amount of Sp1-targeted siRNA or control siRNA. [file 12860_2020_292_MOESM3_ESM.tif]

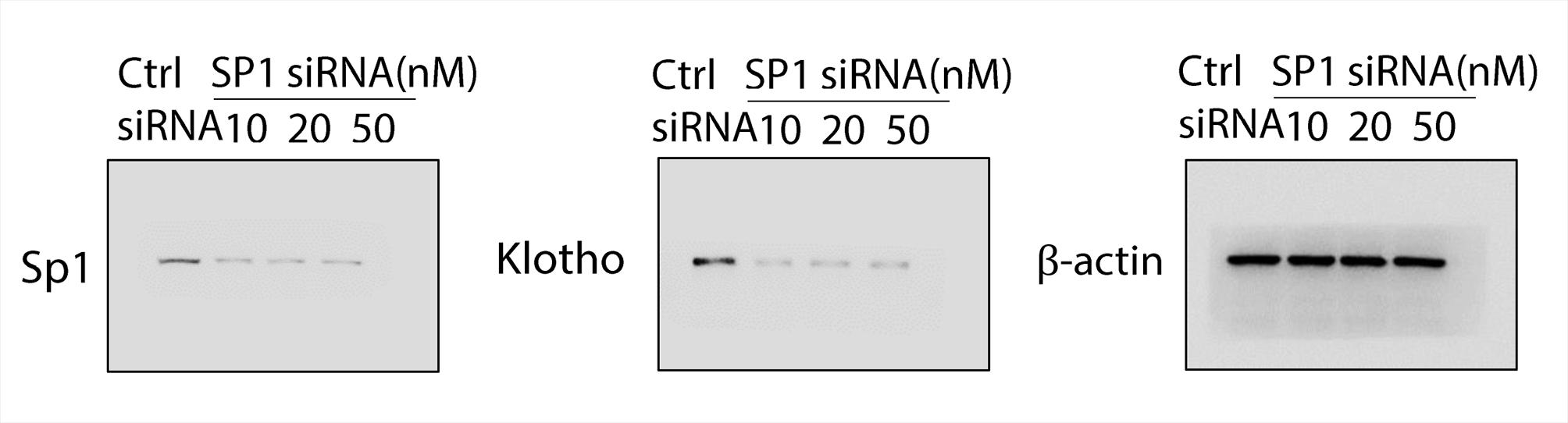

Supplement: Supplementary file 4 — Additional file 4. Western blots in Fig. 2c. The original Western blot images of Sp1、Klotho and β-actin in HEK-293 cells transfected with increasing amount of Sp1-targeted siRNA or control siRNA. [file 12860_2020_292_MOESM4_ESM.tif]

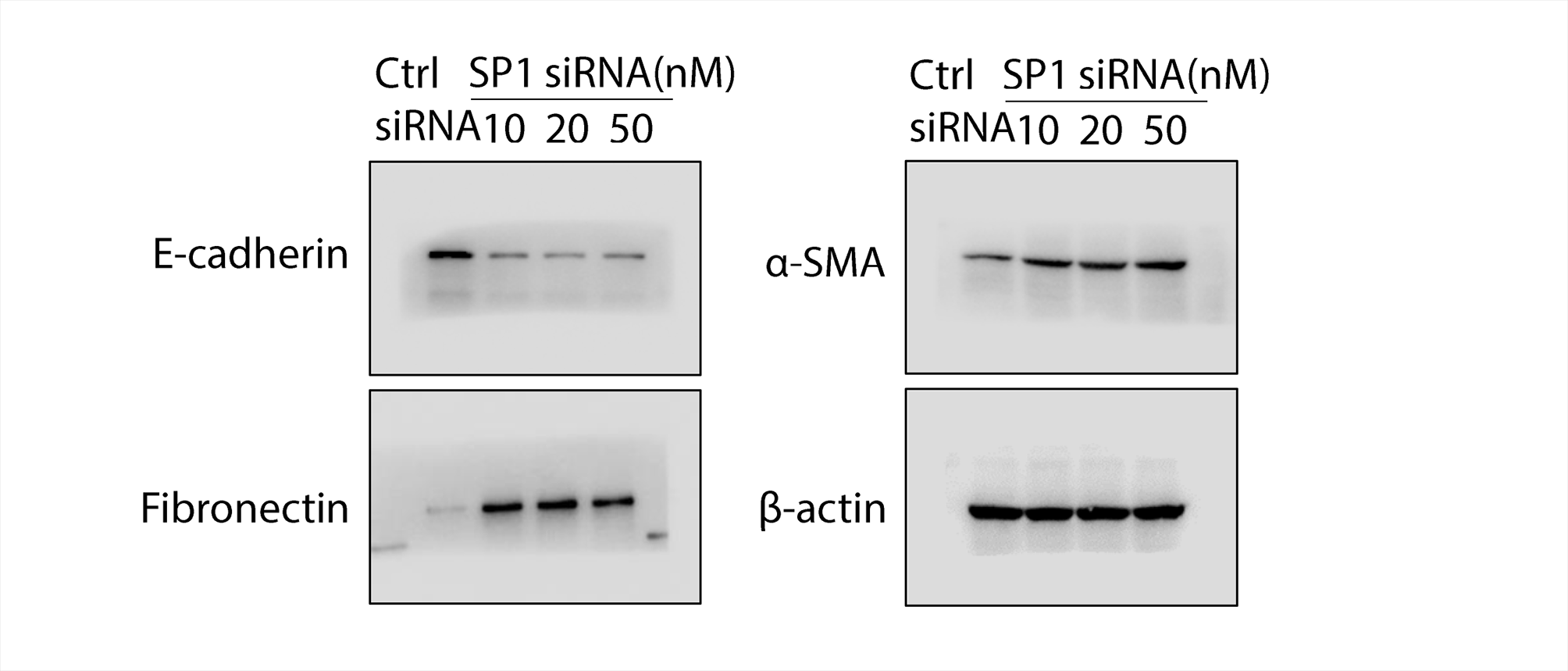

Supplement: Supplementary file 5 — Additional file 5. Western blots in Fig. 2e. The original Western blot images of E-cadherin, α-SMA, Fibronectin and β-actin in HK-2 cells transfected with increasing amount of Sp1-targeted siRNA or control siRNA. [file 12860_2020_292_MOESM5_ESM.tif]

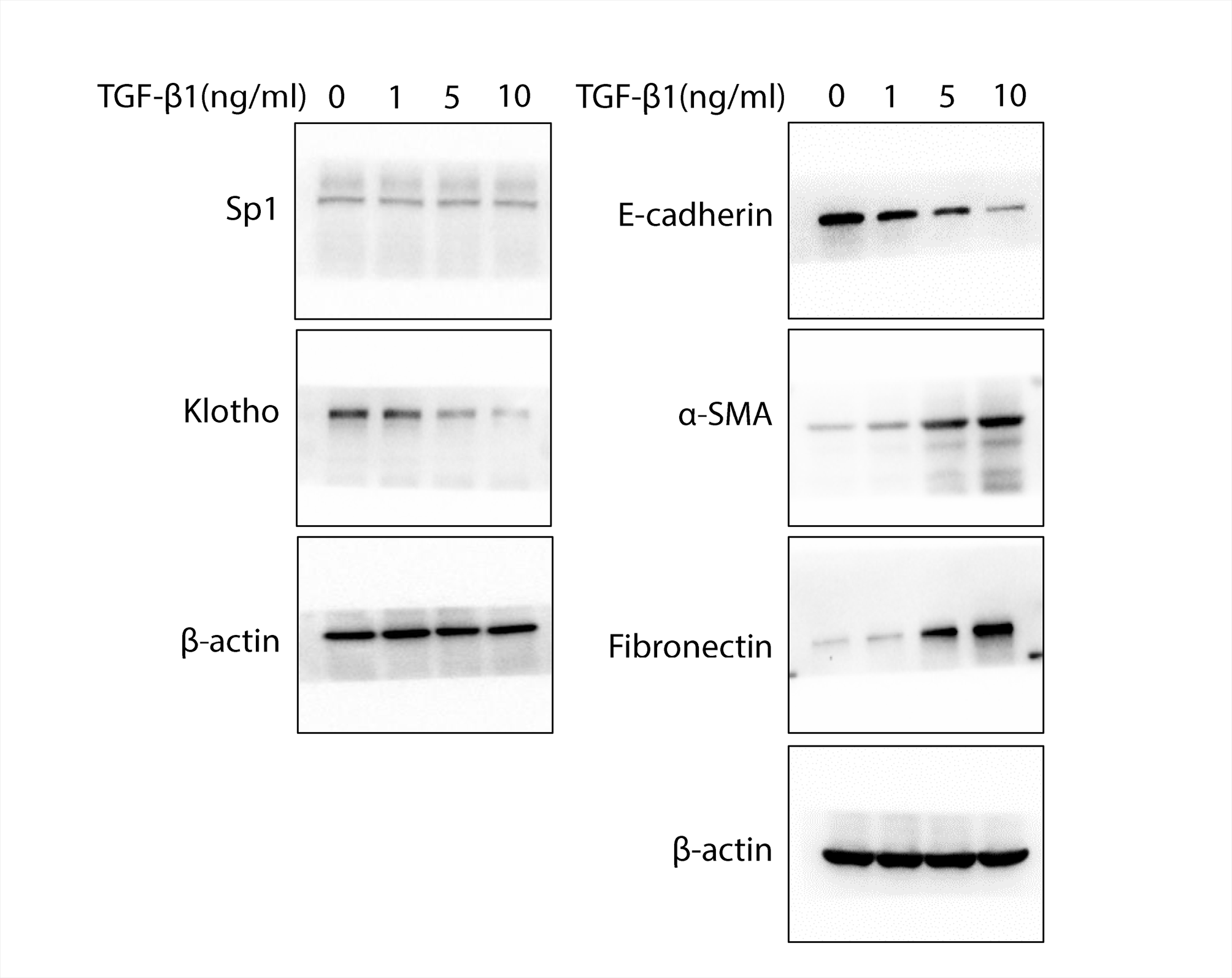

Supplement: Supplementary file 6 — Additional file 6. Western blots in Fig. 4a, b. The original Western blot images of Sp1、Klotho, β-actin, E-cadherin, α-SMA, Fibronectin and β-actin in HK-2 cells treated with increasing amount of TGF-β1. [file 12860_2020_292_MOESM6_ESM.tif]

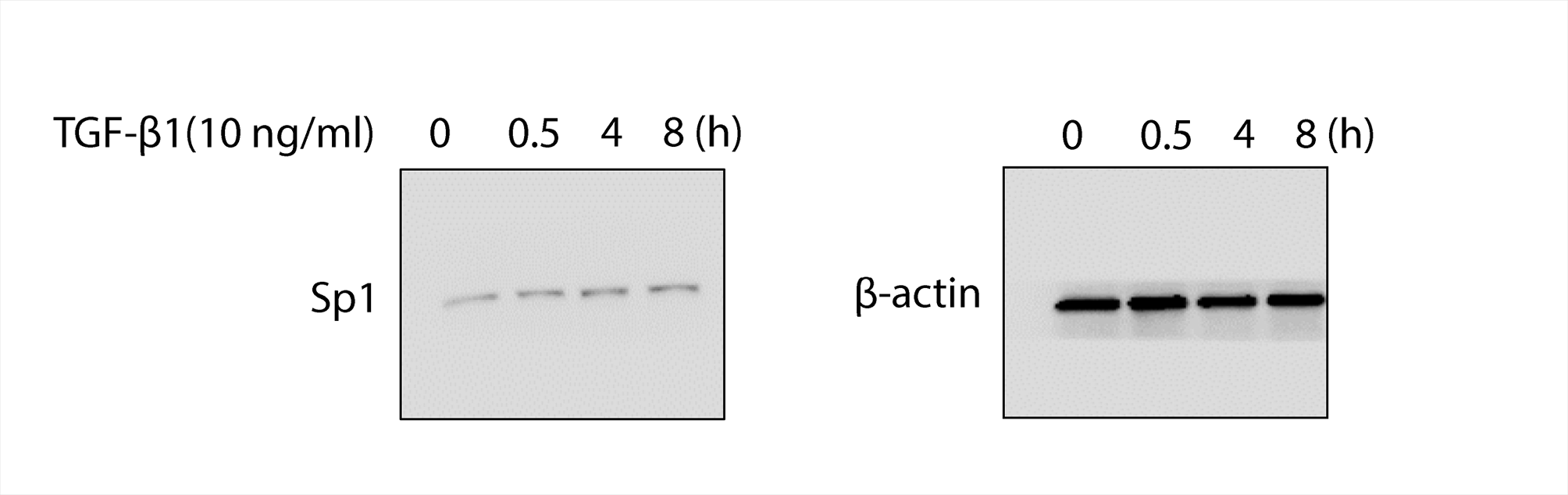

Supplement: Supplementary file 7 — Additional file 7. Western blots in Fig. 4c. The original Western blot images of Sp1 and β-actin in HK-2 cells treated with TGF-β1(10 ng/mL) for 0.5, 4, 8 h. [file 12860_2020_292_MOESM7_ESM.tif]

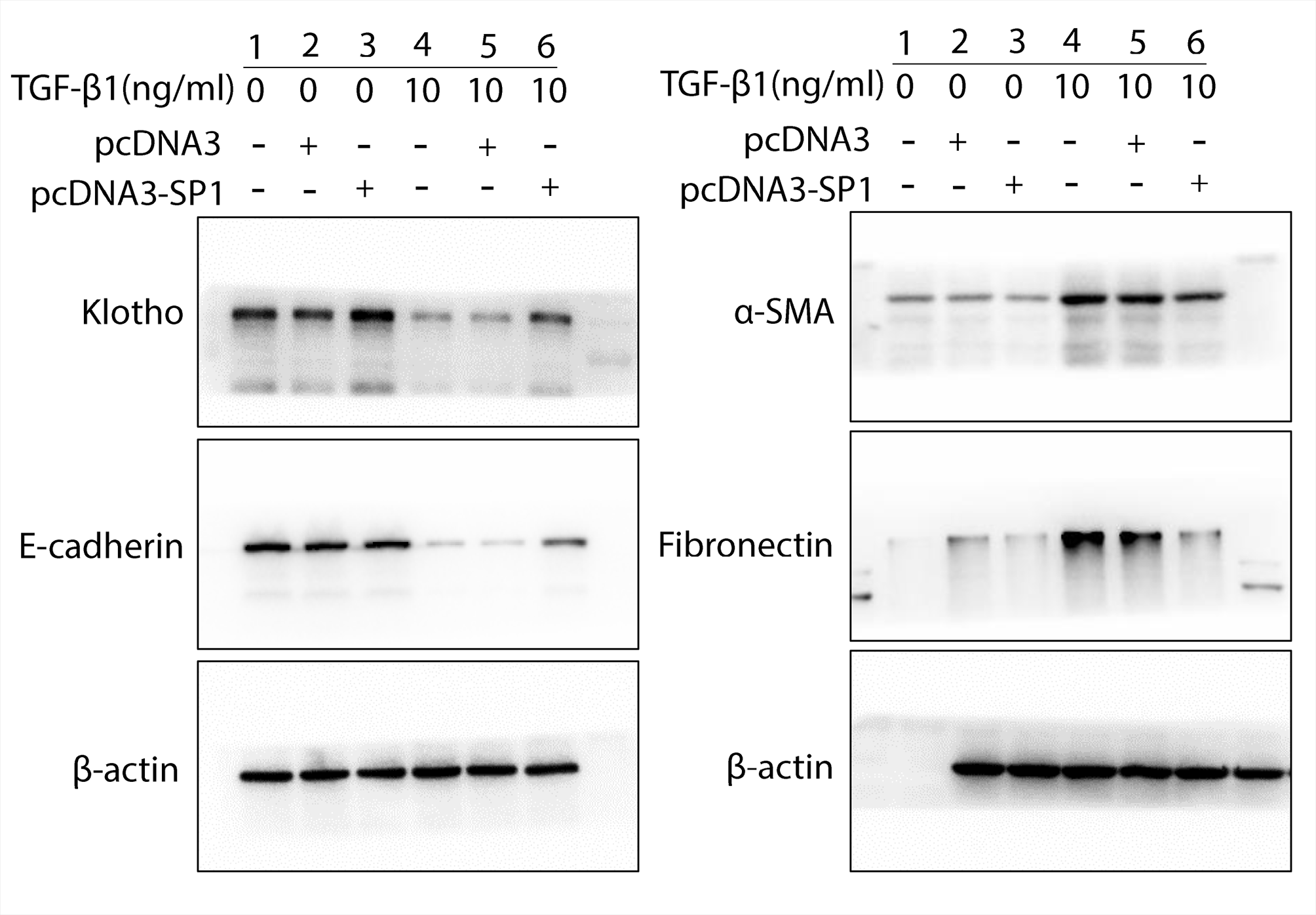

Supplement: Supplementary file 8 — Additional file 8. Western blots in Fig.4f. The original Western blot images of Klotho, E-cadherin, β-actin, α-SMA, Fibronectin and β-actin in HK-2 cells transfected with pcDNA3-Sp1 plasmid or empty vector followed by treating with TGF-β1. [file 12860_2020_292_MOESM8_ESM.tif]

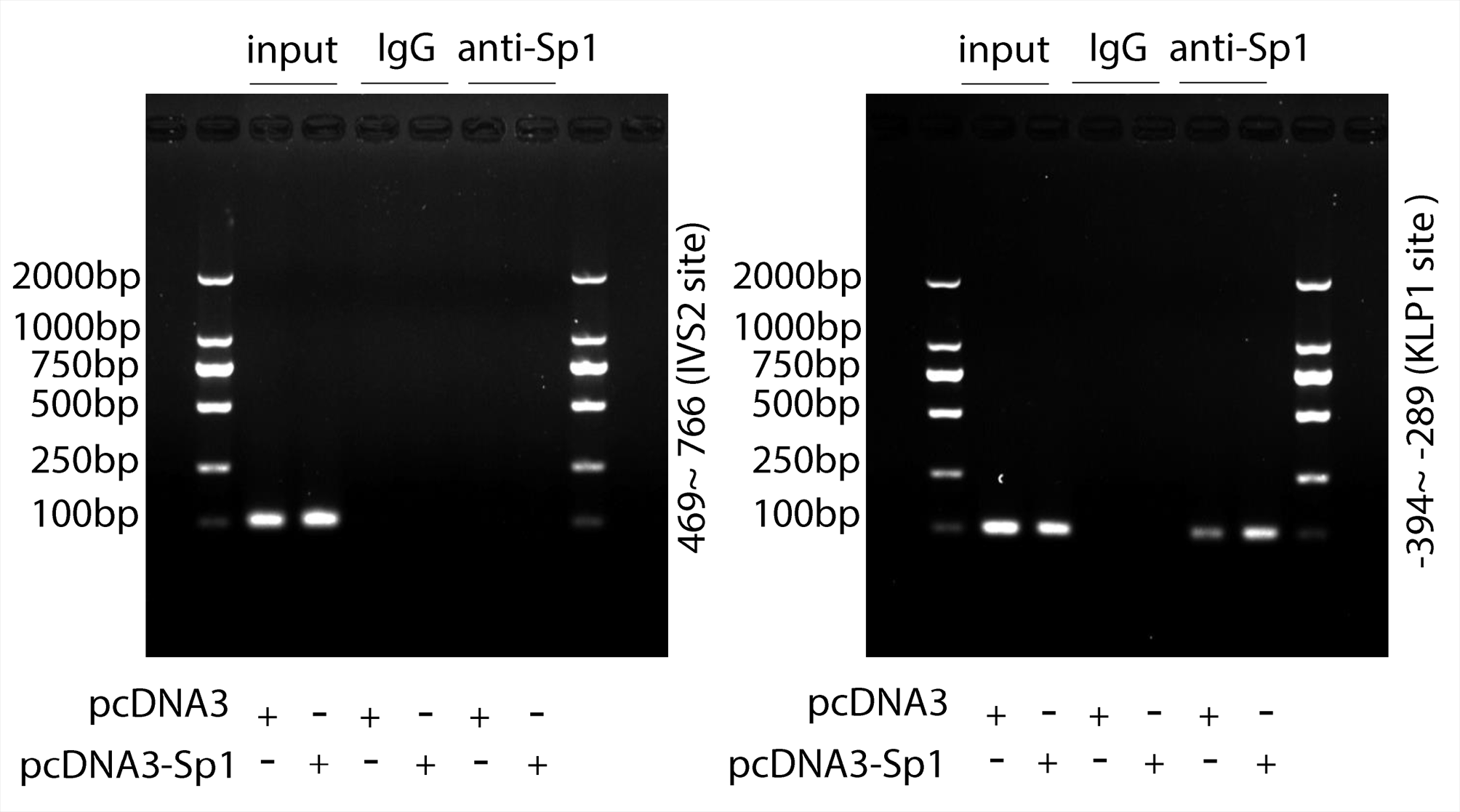

Supplement: Supplementary file 9 — Additional file 9. ChIP assay in Fig. 6b. The original images of ChIP assay in HK-2 cells transfected with pcDNA3-Sp1 plasmid or empty vector. [file 12860_2020_292_MOESM9_ESM.tif]
